# Sleep Duration and Weight Gain: Reconsideration by Panel Data Analysis

**DOI:** 10.2188/jea.JE20140012

**Published:** 2014-09-05

**Authors:** Chihiro Nishiura, Hideki Hashimoto

**Affiliations:** 1 Department of Safety and Health, Tokyo Gas Co., Ltd., Tokyo, Japan; 1 東京ガス株式会社 安全健康・福利室; 2 Department of Health and Social Behavior, School of Public Health, The University of Tokyo, Tokyo, Japan; 2 東京大学大学院 医学系研究科 保健社会行動学分野

**Keywords:** longitudinal studies, sleep, obesity, weight gain, panel analysis

## Abstract

**Background:**

Inconsistent findings in previous studies of the association between sleep duration and changes in body mass index (BMI) may be attributed to misclassification of sleep duration fluctuations over time and unmeasured confounders such as genetic factors. The aim of the present study was to overcome these failings by using repeated measurements and panel data analysis to examine the sleep-BMI association.

**Methods:**

Panel data were derived by secondary use of the data from mandatory health checkups at a Japanese gas company. Male non-shift workers aged 19–39 years in 2007 were annually followed until 2010 (*n* = 1687, 6748 records). BMI was objectively measured, and sleep duration was self-reported.

**Results:**

Compared with 7-hour sleepers, panel analysis with the population-averaged model showed a significant increase in BMI among 5-hour (0.11 kg/m^2^, *P* = 0.001), 6-hour (0.07 kg/m^2^, *P* = 0.038), and ≥8-hour (0.19 kg/m^2^, *P* = 0.009) sleepers. On the other hand, after adjustment for unobserved time-invariant confounders using the fixed-effects model, the magnitude of the association was considerably attenuated and no longer significant (5-hour, 0.07 kg/m^2^, *P* = 0.168; 6-hour, 0.02 kg/m^2^, *P* = 0.631; ≥8-hour sleepers, 0.06 kg/m^2^, *P* = 0.460).

**Conclusions:**

The longitudinal association between sleep duration and changes in BMI may be upwardly biased by unobserved time-invariant confounders rather than misclassified sleep duration. The net effect of sleep duration on weight gain may therefore be less than previously believed.

## Abstract (ja)

**背景：:**

先行研究では睡眠時間とBMI変化の関係は一貫していない。これは睡眠時間の経時的変動の誤分類や、遺伝的要因のような未測定の交絡因子が原因の可能性がある。本研究の目的は、繰り返し測定データを使用したパネル分析により、先行研究におけるこれらの限界を克服して、前述の関連を検討することにある。

**方法：:**

パネルデータは、ある日本のガス会社の法定健康診断データの二次利用により得た。2007年に19-39歳の交替制勤務ではない男性を、2010年まで毎年追跡した（1687名、6748レコード）。BMIは客観的に測定し、睡眠時間は自己申告により測定した。

**結果：:**

7時間睡眠者と比較すると、population-averaged modelによるパネルデータ分析では、5時間睡眠者 (0.11 kg/m^2^, *p *= 0.001)、6時間睡眠者(0.07 kg/m^2^, *p* = 0.038)、8時間以上睡眠者(0.19 kg/m^2^, *p *= 0.009)で、BMIが有意に増加していた。一方、fixed-effects modelにより経時変化しない未観察の交絡要因を調整すると、関連は大きく減弱した(5時間睡眠、0.07 kg/m^2^, *p* = 0.168; 6時間睡眠、 0.02 kg/m^2^, *p* = 0.631; 8時間以上睡眠、0.06 kg/m^2^, *p* = 0.460)。

**結論：:**

睡眠時間とBMI変化の縦断関係は、睡眠時間の誤分類よりも、経時変化しない未観察の交絡因子によって上方に偏っていた可能性がある。睡眠時間が体重増加に与える正味の効果は、従来考えられていたよりも小さいかもしれない。

## INTRODUCTION

An increasing number of longitudinal studies have examined the relationship between sleep duration and weight gain, but they have so far produced mixed results^1^^,^^2^—even among studies limited to young adult subjects, in whom this association is believed to be strongest.^3^^–^^9^ This inconsistency is often attributed to study design limitations. First, previous studies assumed sleep duration and related confounding conditions (e.g. smoking behavior) to be relatively constant for a particular subject and used baseline values to make predictions for weight change over a number of years.^3^^–^^9^ However, intra-subject change has to be taken into account to prevent bias due to potential misclassification of sleep duration. Second—and more serious—is the unobserved confounder issue: recent reviews have identified the presence of a number of possible confounders, including physical activity, nutrition intake and eating habits, sleep disturbance, socioeconomic status and related lifestyle, genetics, and cultural norms regarding body image,^1^^,^^2^^,^^10^ with previous studies including only a limited set of confounder measures. Among these factors, genetic characteristics are a plausible confounder, since recent studies have suggested genetically determined obesogenic mechanisms among short sleepers.^11^^,^^12^ Many previous studies^3^^–^^9^ failed to account for genetic influences, simply relying on a standard regression model in their analysis, which may have been susceptible to residual confounding by unobserved properties such as genetic factors.

Panel data analysis is one way of overcoming the limitations of previous studies, as this method can explicitly model within-subject changes in sleep duration as well as confounders among different survey waves. Additionally, panel data analysis using a fixed-effects model can statistically eliminate the influence of time-invariant unobserved confounders, such as genetic factors.^13^

In this study, we aimed to examine whether a longitudinal association between sleep duration and changes in body mass index (BMI) remains when using panel data and a modeling technique to account for within-subject changes in sleep duration and measured confounders. We also aimed to investigate the influence of unobserved time-invariant confounders.

## METHODS

### Study population

This study was performed using secondary data from legally mandated annual health checkups, which were conducted at a gas company in Tokyo, Japan, for the 2007 to 2010 fiscal years. The participation rate for the checkups was 100%. The checkup month was scheduled according to the birth month of each subject. Subjects enrolled at the 2007 wave (baseline) were annually followed through the 2010 wave.

Since recent reviews have determined that the effect of sleep duration on changes in BMI declines with age in adults,^1^^,^^2^ we studied young adults aged 19–39 years at baseline. Exclusion criteria were as follows: (1) women (*n* = 531), because of the small available sample size; (2) shift workers (*n* = 289), to avoid misclassifying sleep duration, the effect of disturbed circadian rhythms, and disrupted sleep quality due to shift working; (3) subjects for whom any of the follow-up data were lost through leaving the company or being transferred to other work sites (*n* = 140); and (4) subjects for whom any of the sleep duration data were missing (*n* = 152). The final dataset consisted of 1687 subjects with a total of 6748 observation records, and the panel was strongly balanced.

### Dependent variables

At each annual checkup, height and weight were measured by trained health professionals. BMI was calculated as the weight in kilograms divided by the square of the height in meters (kg/m^2^). Changes in BMI were defined as the difference in BMI between the waves.

### Independent variables

All independent variables except for age were collected annually by trained health professionals using a questionnaire survey at each annual checkup. Age was obtained from the company’s administrative records. The data for sleep duration were collected as a continuous variable by asking: “On average, how many hours did you sleep each night over the past month?” The response was categorized into five groups: 4 hours or less, 5 hours, 6 hours, 7 hours, and 8 hours or more. For comparison with previous studies,^3^^–^^9^ the reference category of sleep duration was set at 7 hours.

Other covariates included the following: use of psychiatric medications (yes or no); smoking status (current smoker or not); alcohol consumption, which was obtained as a continuous variable (g/week) and categorized into three groups (0 g/week, 1–139 g/week, ≥140 g/week); habitual leisure time exercise (yes or no), which was defined as exercising one or more times per week; and self-reported symptoms over the past 1 month (sleep disturbance, midmorning sleepiness, midmorning fatigue, ill health, depressive mood, and loss of interest), which was measured using a three-level response and dichotomized (1 = always, 0 = sometimes or never) for analysis. For example, depressive mood was assessed by asking: “On average, how often did you feel in a depressed mood over the past month?” and loss of interest was assessed by asking: “On average, how often did you feel a loss of interest over the past month?” Depressive symptoms were identified as a positive response to either of the two questions relating to a depressive mood and loss of interest.

### Statistical analysis

First, the change trends in the characteristics of the subjects over different waves were examined using generalized estimating equations (GEE), with each variable as a dependent variable and waves as a continuous independent variable to account for the repeated measurements. Differences in alcohol intake were examined by means of a negative binomial link function to include non-drinkers. Second, baseline differences among the sleep duration categories were assessed using analysis of variance for means and the chi-square test for proportions. Third, the 1-year relationships between sleep duration and changes in BMI were examined by panel data analysis and adjusted for all covariates using 5061 records (1687 subjects × 3 years). Therefore, we allowed all explanatory variables, including sleep duration, to change within the same subject from one wave to the next.

We conducted panel data analysis using the population-averaged model by the GEE estimator and the fixed-effects model by the within estimator. Both models were able to account for intra-subject changes in repeated measurements over a wave. The difference was that, whereas the population-averaged model assumes independence between measured explanatory variables with error terms, the fixed-effects model explicitly allows non-zero covariance between explanatory variables and time-invariant unobserved factors.^14^ Any difference in the estimated coefficients between the two models would indicate that residual confounding was not insignificant, and the fixed-effects model should provide a less biased estimation. The difference could be examined using the Hausman test. We initially used the population-average model and then compared the results with those of the fixed-effects model.

Owing to a substantial number of missing values, we conducted multiple imputation using chained equations for the following variables (figures in parentheses indicate the number of missing observations): smoking (5), weekly alcohol intake (8), exercise habit (7), sleep disturbance (13), midmorning sleepiness (14), midmorning fatigue (12), ill health (14), and depressive symptoms (28). In addition to the above variables, the imputation model included the following measurements, which were available among the checkup data: wave year, date of checkup, age, BMI, diastolic blood pressure, low-density lipoprotein cholesterol, and hemoglobin A_1c_. The distributions of all variables were visually similar for observed and imputed data, which indicated that there were no obvious problems with the imputation process (data available upon request). Since the estimated results based on the non-imputed dataset were similar to the estimated results based on the imputed dataset, we present only the results relating to the latter. All statistical calculations were performed using Stata 12 (Stata Corporation, College Station, TX, USA).

### Ethical approval

Written consent for generic use of health checkup data for research purposes was obtained from all participants at the time of the checkups. No participant requested that their data not be used for secondary research purposes. The gas company approved the secondary use of the health checkup data for this study. All data were provided anonymously. The study was approved by the ethical committee of the University of Tokyo (review no. 10440).

## RESULTS

The characteristics of the subjects in each wave are summarized in Table 1. Mean BMI, the prevalence of obesity, and mean alcohol intake gradually increased, and mean sleep duration and the prevalence of smoking decreased over successive waves. The proportions of exercise habits, use of psychiatric medication, and self-reported symptoms did not differ among the waves.

**Table 1.** Descriptive statistics of the panel data^a^

	Waves				*P* value^b^

	2007	2008	2009	2010	
Number of subjects	1687	1687	1687	1687	
Age (years)	33.4 (5.0)	34.4 (5.0)	35.4 (5.0)	36.5 (5.0)	0.000
BMI (kg/m^2^)	23.5 (3.3)	23.5 (3.2)	23.6 (3.3)	23.7 (3.4)	0.000
Obesity (BMI of ≥25.0 kg/m^2^) (%)	26.6	26.2	28.2	29.4	0.000
Sleep duration (hours)	5.93 (0.82)	5.92 (0.82)	5.88 (0.85)	5.89 (0.83)	0.002
Drinking habit (%)	84.4	85.5	86.4	86.1	0.001
Alcohol (g/week)	114.6 (116.9)	125.7 (127.3)	128.2 (130.9)	130.6 (125.6)	0.000
Exercise habit (%)	48.1	47.3	47.1	46.9	0.288
Smoking habit (%)	40.8	39.9	39.7	37.8	0.000
Psychiatric medication (%)	0.2	0.3	0.2	0.2	0.896
Depressive symptoms (%)	5.3	5.0	5.1	4.9	0.579
Ill health (%)	2.5	2.3	2.0	2.4	0.731
Sleep disturbance (%)	3.2	2.9	2.7	2.5	0.159
Midmorning sleepiness (%)	3.6	3.8	3.6	3.0	0.285
Midmorning fatigue (%)	4.5	5.0	4.9	4.2	0.511

BMI, body mass index.

^a^Data are expressed as means (standard deviation) or percentages. Each percentage was calculated without missing data.

^b^Change trends in the characteristics of the subjects among the different waves were examined using generalized estimating equations with each variable as a dependent variable and waves as a continuous independent variable to take into account the repeated measurements.

Table 2 shows the cross-sectional characteristics of the subjects according to sleep duration category at the first wave in 2007. Mean BMI and the prevalence of obesity were lowest among the participants who slept 6 hours. Mean alcohol intake was higher among participants who slept 7 hours or more. The proportion of subjects who habitually exercised was greater in the longer sleep duration categories. The prevalence of depressive symptoms was higher among participants who slept 5 hours or less. The symptoms of sleep disturbance, midmorning sleepiness, and midmorning fatigue were more frequently observed among short sleepers.

**Table 2.** Characteristics of subjects in the 2007 wave according to sleep duration category^a^ (*n* = 1687)

	Overall	Self-reported sleep duration at the 2007 wave (hours)					*P* value^b^

		≤4	5	6	7	≥8	
Number of subjects (% of overall)	1687 (100)	66 (3.9)	448 (26.6)	852 (50.5)	277 (16.4)	44 (2.6)	
Age (years)	33.4 (5.0)	33.1 (4.6)	33.4 (5.2)	33.4 (4.9)	33.8 (5.0)	32.7 (5.0)	0.550
BMI (kg/m^2^)	23.5 (3.3)	24.2 (3.8)	24.0 (3.7)	23.2 (3.0)	23.3 (3.0)	23.6 (2.8)	0.000
Obesity (BMI of ≥25.0 kg/m^2^) (%)	26.6	33.3	33.5	22.9	24.2	34.1	0.000
Drinking habit (%)	84.4	90.9	82.1	85.6	83.0	81.8	0.255
Alcohol (g/week)	114.6 (116.9)	117.3 (100.9)	109.9 (114.2)	112.1 (114.8)	126.4 (127.4)	133.2 (136.1)	0.000
Exercise habit (%)	48.1	36.9	47.0	47.1	54.4	57.1	0.050
Smoking habit (%)	40.8	43.9	44.6	38.6	40.4	41.9	0.316
Psychiatric medication (%)	0.2	0.0	0.0	0.1	0.7	0.0	0.215
Depressive symptoms (%)	5.3	7.6	9.0	3.8	4.0	2.3	0.001
Ill health (%)	2.5	4.6	3.6	2.0	1.4	4.6	0.187
Sleep disturbance (%)	3.2	10.8	4.7	2.1	2.5	0.0	0.000
Midmorning sleepiness (%)	3.6	10.6	5.2	2.8	2.2	0.0	0.002
Midmorning fatigue (%)	4.5	9.1	6.7	3.4	3.6	2.3	0.020

BMI, body mass index.

^a^Data are expressed as means (standard deviation) or percentages. Each percentage was calculated without missing data.

^b^*P* value: comparison across waves using analysis of variance for means and the chi-square test for proportions.

Table 3 presents the results of panel data analysis. Compared with 7-hour sleepers, panel analysis with the population-averaged model showed that participants who slept 5, 6, and 8 or more hours per night had significant gains in BMI: 0.11 kg/m^2^ (95% confidence interval (CI) 0.05–0.18) for those who slept 5 hours per night; 0.07 kg/m^2^ (95% CI 0.00–0.13) for those who slept 6 hours per night; and 0.19 kg/m^2^ (95% CI 0.05–0.33) for those who slept 8 or more hours per night. On the other hand, the results in the fixed-effects model did not reach statistical significance, with a remarkable attenuation of changes in BMI: 0.07 kg/m^2^ (95% CI −0.03–0.18) for those who slept 5 hours per night; 0.02 kg/m^2^ (95% CI −0.06–0.11) for those who slept 6 hours per night; and 0.06 kg/m^2^ (95% CI −0.11–0.24) for those who slept 8 or more hours per night. The *P* value for the Hausman test was <0.001, which indicates that the fixed-effects model provided a less biased estimate than the population-averaged model.

**Table 3.** Results of the longitudinal relationship between sleep duration and BMI using panel data analysis (*n* = 1687, 5061 records)

Variables on models	1-year changes in BMI (kg/m^2^)			

	Population-averaged model		Fixed-effects model	
	
	B	95% CI	B	95% CI
Sleep duration				
≤4 hours	0.05	(−0.07, 0.17)	0.08	(−0.08, 0.25)
5 hours	0.11	(0.05, 0.18)	0.07	(−0.03, 0.18)
6 hours	0.07	(0.00, 0.13)	0.02	(−0.06, 0.11)
7 hours	0.00	(Reference)	0.00	(Reference)
≥8 hours	0.19	(0.05, 0.33)	0.06	(−0.11, 0.24)
Exercise (reference, none)				
Regular exerciser	−0.01	(−0.06, 0.03)	−0.13	(−0.20, −0.06)
Smoking (reference, non-smoker)				
Smoker	0.07	(0.03, 0.11)	0.05	(−0.08, 0.19)
Alcohol consumption (reference, 0 g/week)				
1–139 g/week	0.00	(−0.06, 0.06)	−0.01	(−0.14, 0.12)
≥140 g/week	0.00	(−0.07, 0.06)	−0.03	(−0.18, 0.12)
Psychiatric medications (reference, none)	0.22	(−0.24, 0.67)	−0.02	(−0.55, 0.51)
Symptoms (reference, none)				
Depression	0.13	(0.02, 0.24)	0.12	(0.00, 0.25)
Ill health	−0.01	(−0.17, 0.16)	0.06	(−0.13, 0.24)
Sleep disturbance	0.03	(−0.11, 0.16)	0.00	(−0.16, 0.17)
Midmorning sleepiness	0.00	(−0.12, 0.13)	0.10	(−0.05, 0.25)
Midmorning fatigue	−0.01	(−0.13, 0.10)	−0.12	(−0.26, 0.02)
Age (years)	0.00	(−0.01, 0.00)	0.12	(0.10, 0.14)
BMI (kg/m^2^)	−0.01	(−0.02, −0.01)	−1.01	(−1.04, −0.98)

B, beta coefficient; BMI, body mass index; CI, confidence interval.

## DISCUSSION

In this study, in order to overcome the limitations of previous studies, we examined the association between sleep duration and changes in BMI by means of panel data analysis. Both the population-averaged and fixed-effects models incorporated within-subject changes in sleep duration and confounders among the different survey waves. The population-averaged model, which assumes the absence of covariance with unobserved confounders, detected a significant association between sleep and BMI. Conversely, the fixed-effects model, which explicitly accounts for time-invariant unobserved confounders, showed a largely attenuated association between sleep and BMI. A comparison of the two models suggests that the previously identified association between sleep and BMI may have been overestimated due to residual confounding through time-invariant unobserved confounders such as genetic properties.

Although Table 2 showed the cross-sectional relation of sleep duration with BMI, alcohol consumption, exercise habit, depressive symptoms, sleep disturbance, midmorning sleepiness, and midmorning fatigue, the results of the population-averaged model in Table 3 indicated that sleep duration was associated with subsequent changes in BMI after adjustment for the above-mentioned possible confounders.

There are a large number of possible confounders that previous studies and the current study could not fully measure. Of them, we mathematically eliminated the effect of time-invariant unobserved confounders from the estimates using a fixed-effects model and found that unobserved time-invariant confounders had a large impact on the sleep-BMI association. One likely time-invariant confounder is genetics. Prats-Puig et al conducted a genetic study of 297 asymptomatic children aged 5–9 years^11^ and reported that genetic variations in the obesity genes FTO, TMEM18, and NRXN3 can modulate the vulnerability of children to weight gain related to short sleep duration. Watson et al conducted a statistical study of 1088 pairs of twins in a population-based sample in the United States.^12^ Using structural equation model analysis, they determined that shorter sleep duration increases the expression of genetic factors for greater body weight. These studies suggest a correlation between sleep duration and genetic disposition. Further, some possible confounders proposed by previous papers appear to have a time-invariant nature, e.g. social, environmental, and parental factors.^2^^,^^10^

Physical activity is a major possible confounder of the sleep-BMI association. In our results, exercise habit was not significant in the population-averaged model but was significant in the fixed-effects model. Exercise habit is known to be a relatively constant behavior,^15^^,^^16^ and its impact should therefore be eliminated in the fixed-effects model. Thus, the effect of habitual exercise on BMI may be attributed to changes in exercise habits over time rather than constant exercise habits.

The estimated associations in the population-averaged model in our sample indicate a greater magnitude for the association between short sleep and weight gain (beta coefficient, 0.11 kg/m^2^ over 1 year among participants who slept 5 hours) than that found in previous Japanese studies.^4^^,^^17^^–^^19^ Among those studies, two were conducted at the same workplace as in the present study, though they used a different time frame and the participants were older than in the present study.^17^^,^^18^ The relatively young age of the subjects and the settings used in the present study may therefore have resulted in a more conspicuous association between sleep and BMI.

Although it was not statistically significant, the observed association between sleep and BMI in the fixed-effects model may be explained by biological mechanisms that involve time-variant factors, such as mechanisms via leptin,^20^ and obesogenic behavioral mechanisms that entail consuming more calories and engaging in less physical activity as a result of short-sleep lifestyles. The existence of such causal mechanisms needs to be examined using larger datasets.

The strengths of the present study include the following: the extremely high response rate, which reduced selection bias related to dropouts; the use of an occupational cohort, which avoided the inclusion of subjects who were unable to work due to health reasons; and the homogenous study population through sampling from a single company, which was less susceptible to such socioeconomic confounding factors as income and education.

In terms of study limitations, the first is that the reliability and validity of the self-reported variables, such as depression symptoms, were unclear, which may have resulted in insufficient adjustment. Second, our measure of sleep duration was based on self-reporting over the previous month. Actigraphy is currently the best measurement of sleep duration for epidemiological studies; however, we were unable to use actigraphy owing to the large sample size. Self-reported sleep duration may be systematically longer than actigraphy-measured sleep.^21^^,^^22^ Third, there was a lack of information about dietary intake. Although the fixed-effects model should eliminate the effect of time-invariant unobserved dietary intake, time-variant dietary intake was not adjusted in our results. Fourth, there may be seasonal variation in diet, physical activity, body weight and sleep, which may bias the results if participants had checkups in different seasons at different waves. However, in this study, within-subject difference in actual checkup date between waves was 2.1 days (standard deviation 45.5 days), and 95% of subjects had their checkups roughly 9–15 months after their previous checkup date. Therefore, our results in the fixed-effects model treated any potential seasonal effect as a time-invariant factor within subjects, and the model should eliminate the effect from the estimates.

In conclusion, the longitudinal association between sleep duration and changes in BMI may be upwardly biased by unobserved time-invariant confounders, rather than within-subject changes in sleep duration over time. The net effect of sleep duration on weight gain may therefore be less than previously believed. Further research is necessary to assess whether short sleep duration can be a risk factor for weight gain.

## ONLINE ONLY MATERIAL

Abstract in Japanese.
