# Supplementary material for: Sleep Duration and Weight Gain: Reconsideration by Panel Data Analysis
Source: J Epidemiol. 2014 Sep 5;24(5):404–9. doi: 10.2188/jea.JE20140012 (PMC4150012; doi:10.2188/jea.JE20140012)
Supplement: Abstract in Japanese. [file je-24-404-s001.pdf]

タイトル: 睡眠時間と体重増加：パネルデータ分析による再検討

著者: 西浦千尋[1]、 橋本英樹[2]

著者所属:

[1] 東京ガス株式会社　安全健康・福利室

[2] 東京大学大学院　医学系研究科　保健社会行動学分野

要約

背景: 先行研究では睡眠時間と BMI 変化の関係は一貫していない。これは睡眠時間の経時的変動の誤分類や、遺伝的要因のような未測定の交絡因子が原因の可能性がある。本研究の目的は、繰り返し測定データを使用したパネル分析により、先行研究におけるこれらの限界を克服して、前述の関連を検討することにある。

方法: パネルデータは、ある日本のガス会社の法定健康診断データの二次利用により得た。2007 年に 19-39 歳の交替制勤務ではない男性を、2010 年まで毎年追跡した（1687 名、6748 レコード）。BMI は客観的に測定し、睡眠時間は自己申告により測定した。

結果: 7 時間睡眠者と比較すると、population-averaged model によるパネルデータ分析では、5 時間睡眠者 (0.11 kg/m^2, p = 0.001)、6 時間睡眠者(0.07 kg/m^2, p = 0.038)、8 時間以上睡眠者(0.19 kg/m^2, p = 0.009)で、BMI が有意に増加していた。一方、fixed-effects model により経時変化しない未観察の交絡要因を調整すると、関連は大きく減弱した(5 時間睡眠、0.07 kg/m^2, p = 0.168; 6 時間睡眠、 0.02 kg/m^2, p = 0.631; 8 時間以上睡眠、0.06 kg/m^2, p = 0.460)。

結論: 睡眠時間と BMI 変化の縦断関係は、睡眠時間の誤分類よりも、経時変化しない未観察の交絡因子によって上方に偏っていた可能性がある。睡眠時間が体重増加に与える正味の効果は、従来考えられていたよりも小さいかもしれない。

キーワード: 縦断研究, 睡眠, 肥満, 体重増加, パネル分析
